# Breast Cancer and Body Image Issues

**Published:** 2018-05-01

**Authors:** Pamela Hallquist Viale

Breast cancer remains a common malignancy, with an estimated 366,120 new cases expected in women in the United States this year (Breastcancer.org, 2018). Although the incidence rate for this disease has decreased since the year 2000, the disease remains deadly for some: Approximately 40,920 women in the US are expected to die from breast cancer this year. It is frightening to receive a breast cancer diagnosis, but overall breast cancer death rates have declined secondary to improvements in treatment and early detection (American Cancer Society, 2017).

That’s good news for women facing the disease, but even successful treatment for breast cancer can cause depression and psychological distress. Changes in body image can produce significant distress as patients experience treatment-related alopecia, loss of eyebrows or eyelashes, or mastectomy (Pierrisnard et al., 2017). Patients can suffer from infertility, lymphedema, medically induced menopause and vaginal dryness, or impaired sexual functioning. Patients may describe feelings of grief, changes in perceived femininity, and low confidence (Esplen, Wong, Warner, & Toner, 2018). In the course of my career taking care of breast cancer patients, I’ve even encountered the rare person who declined appropriate therapy because of fear of perceived disfigurement.

## TOOLS TO IMPROVE BODY IMAGE

A meta-analysis demonstrated that cognitive behavioral therapy (CBT) can improve the quality of life and psychological health of breast cancer survivors and patients, with the authors recommending CBT as an intervention for patients whenever possible (Ye et al., 2018). A recently published Canadian study by Esplen and colleagues describes a group psychological intervention aimed at an improvement of disturbances in body image (BI), sexual functioning, and quality of life in breast cancer survivors (2018).

In a prospective, randomized controlled trial, researchers assessed the efficacy of a 2-month group intervention in 131 women (with 63 allocated to the control group) who had completed surgery, adjuvant chemotherapy, and radiation therapy. The primary goal of the study was to assess the intervention’s effect on positive BI, with secondary outcomes of sexual functioning and quality of life (Esplen et al., 2018). Patients were followed for a year. Two scales were used to measure BI: the Body Image Scale (BIS), a 10-item single factor scale assessing perceived appearance and changes, and the Body Image After Breast Cancer Questionnaire (BIBCQ). The questionnaire describes additional BI factors; the focus of this published paper was on body stigma and vulnerability. Additional scales assessed sexual functioning and quality of life (Esplen et al., 2018).

Restoring Body Image After Cancer (ReBIC), the intervention developed for the study, incorporates guided imagery using well-documented group-therapy principles to assist with difficulties with BI and/or sexual functioning. The group met in a safe and trusting environment, discussing BI issues such as self-image embarrassment, lack of trust in body functioning, and avoidance of sexual activity/intimacy (Esplen et al., 2018). Women participants expressed feeling that their bodies had let them down or that they felt unattractive. These feelings were present regardless of the type of surgical intervention or reconstructive surgery techniques.

## RESULTS OF REBIC INTERVENTION

The primary outcomes demonstrated a significant change in BI by the 12-month follow-up, with participants reporting lower levels of BI concerns compared with the control group (F[1,155] = 6.51; *p* < .01). The changes in body stigma and body vulnerability were significant as well, with the study group demonstrating a lower level of stigma (F[1,162] = 6.52; *p* < .01). The guided imagery part of the group intervention was well received by the participants, helping women to confront their feelings about an altered body and self or assisting the participants to recognize other characteristics of themselves contributing to new self-images (Esplen et al., 2018).

## IMPLICATIONS FOR ADVANCED PRACTITIONERS

Increased recognition of the specific body image issues facing women with breast cancer is important. This can be a life-threatening disease, which is frightening enough. Adding in the very real issues associated with mastectomy, treatment-related complications of alopecia, and changes in sexual functioning, one realizes how overwhelming this disease can be. Advanced practitioners are usually aware of the myriad of issues facing our patients with breast cancer. Recognition of possible interventions to improve the fears and worries of this group of patients is important. Advanced practitioners must also remain aware that in our rapidly changing health-care environment, challenges to the implementation of CBT and BI programs include time, fiscal issues, and training of personnel. It is our responsibility to recognize the concerns our patients with breast cancer face and discover the optimal way to address these issues.

## JADPRO LIVE 2018

Did you hear the news? Registration is open for JADPRO Live, which takes place at The Diplomat hotel in Hollywood, Florida, from November 1 through 4. Visit jadprolive.com to learn more about the meeting and register. I hope to see you there!
